# Mapping of snail intermediate host habitats reveals variability in schistosome and non-schistosome trematode transmission in an endemic setting

**DOI:** 10.1016/j.crpvbd.2025.100299

**Published:** 2025-07-24

**Authors:** Teckla Angelo, Naima Camilla Starkloff, David James Civitello, Moses Paul Mahalila, Safari Kinung’hi

**Affiliations:** a National Institute for Medical Research (NIMR), Mwanza Centre, P.O. Box 1462, Mwanza, Tanzania; b School of Life Sciences and Bioengineering, Nelson Mandela African Institution of Science and Technology, P.O. Box 447, Arusha, Tanzania; c Department of Biology, Emory University, Atlanta, GA, 30322, USA; d Institute for Biodiversity and Ecosystem Dynamics at the University of Amsterdam, Science Park 904, 1098 XH, Amsterdam, Netherlands

**Keywords:** *Bulinus nasutus*, *Schistosoma haematobium*, Habitat, Waterbody, Transmission

## Abstract

The intermediate snail host of *Schistosoma haematobium*, the etiological agent of urogenital schistosomiasis, serves as a critical sentinel for tracking the spread of associated disease risks. In addition to *S. haematobium*, *Bulinus* spp*.* snails also transmit *S. bovis* to cattle as well as several non-schistosome trematodes to cattle and wildlife. Identifying transmission foci of these multi-parasite hosts is critical for targeted and effective One Health intervention. We investigated 467 waterbodies in 86 villages across six districts in northwestern Tanzania. A total of 43,348 *Bulinus nasutus* were collected across three survey phases from November 2020 to August 2021. Across all snails, 0.63% were emitting schistosome cercariae. There was a significant increase in schistosome prevalence during the year, with a peak in the dry season (June-August 2021). Furthermore, of the 25,052 snails collected in the latter two phases (March to August 2021), 4.9% were infected with non-schistosome trematodes, exceeding prevalences of schistosomes at all spatial scales. Co-infections were uncommon, with only 0.05% of snails concurrently emitting both schistosome and non-schistosome parasites. These infection patterns were consistent across village and district levels. Waterbodies used by cattle had higher schistosome prevalence than waterbodies isolated for human use. Surprisingly, non-schistosome prevalence was equal in both of these waterbody types. This suggests that cattle have an indirect role in schistosome transmission, requiring the separation of waterbody usage between cattle and humans and extending snail control in dry season to waterbodies used by cattle. By contrast, water permanence and school proximity did not impact snail or parasite presence. Targeted interventions should focus on local water use dynamics, with attention to the potential indirect role of cattle in schistosome transmission.

## Highlights

• Habitat variability of *Bulinus nasutus* in schistosome and non-schistosome trematode parasite transmission was studied.
• The prevalence of patent schistosome infections in *B. nasutus* snails was low compared to non-schistosome trematodes.
• *Bulinus nasutus* presence and infection status varied greatly at the waterbody level.
• Schistosome prevalence was higher in waterbodies used by cattle than in waterbodies used by humans alone.
• Co-infection with schistosome and non-schistosome trematodes was uncommon, with a prevalence of 0.05%.

## Graphical abstract

**Figure:**
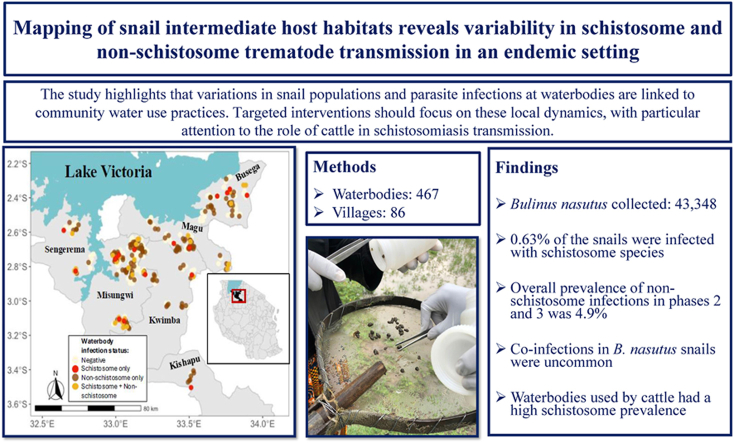

## 1 Introduction

Schistosomiasis is a waterborne parasitic disease that presents major public health challenges in regions where human populations frequently interact with contaminated freshwater environments. The disease is transmitted through contact with water containing *Schistosoma* spp., released by snails infected after exposure to human excreta (Gryseels and Polman, 2006)*.* Snails of the genus *Bulinus* act as intermediate hosts for the trematode *Schistosoma haematobium* and are widely distributed, leading to a high disease distribution in Tanzania (Webbe, 1962; Clements et al., 2008). *Bulinus* spp. snails tend to occupy different types of waterbodies where they transmit *S. haematobium*, a causative agent of urogenital schistosomiasis in humans. Globally, it is estimated that over 112 million people are currently infected with *S. haematobium*, and the majority of sub-Saharan African countries are at risk of infection (Pennington et al., 2017).

In Tanzania, schistosomiasis continues to pose a major health challenge characterized by wide spatial distribution and infection rates of snails that harbor schistosome parasites (Starkloff et al., 2024). Across Tanzania, *Schistosoma haematobium* is widespread, facilitated by extensive habitat suitability for *Bulinus* spp. snail intermediate hosts. *Bulinus* spp. survive in diverse freshwater bodies, including seasonal ponds, wells, rivers, and irrigation channels, driving sustainable parasite transmission even in temporary water sources. Geographically, *S. haematobium* occurs in the inland of the eastern and southeastern hinterland of Lake Victoria (Lwambo, 1988) and lowland areas on the eastern coast of the country in Unguja and Pemba islands (Zanzibar Archipelago) (Blair, 1956; Mazigo et al., 2012; Pennance et al., 2020). The disease exhibits considerable variability, with prevalence ranging from 12.7% to 87.6% in areas where access to water infrastructure and preventive measures is limited (Tanzania Ministry of Health, 2021). Three major *Bulinus* spp. in Tanzania are responsible for *S. haematobium* transmission, *Bulinus africanus*, *Bulinus globosus*, and *B. nasutus* (Clements et al., 2008; Mazigo et al., 2012). Of these, *B. nasutus* is widely distributed throughout the country, occupying different waterbodies ranging from small and temporary to large and permanent, while *B. globosus* is predominantly found in coastal areas (Stothard et al., 2002). *Bulinus africanus* occurs in the southern highland areas in rivers, swamps, and temporary waterbodies (Mandahl-Barth, 1965; Sturrock, 1965; Lwambo, 1988; Stothard et al., 2002; Mazigo et al., 2012). The adaptability of *Bulinus* spp. snails to inhabit diverse water sources significantly increases the likelihood that many waterbodies will harbor these snails, thereby serving as transmission hubs for *S. haematobium*. Identifying these waterbodies as primary transmission hotspots is crucial for implementing targeted strategies to effectively control the spread of urogenital schistosomiasis.

Expanding agricultural activities with irrigation practices around waterbodies drive frequent water contact, leading to schistosome exposure (Huang and Manderson, 1992; Steinmann et al., 2006; Mazigo et al., 2012). Environmental studies on schistosome transmission have been conducted throughout Africa, focusing on environmental factors such as water sources, climate, and land use that contribute to the spread of schistosomiasis (Boelee and Madsen, 2006; Rubaba et al., 2016; Kalinda et al., 2017a; Mereta et al., 2019; Adekiya et al., 2020; Tabo et al., 2024). However, schistosomiasis research in Tanzania has predominantly focused on human infections, with limited attention given to the snail habitats that play a central role in disease transmission. In inland waterbodies, increased schistosome transmission has been linked to the expansion of irrigation farming, rice paddies, and other water-dependent agricultural activities, which create favorable environments for *Bulinus* spp. snails, the intermediate hosts responsible for spreading schistosomiasis (Steinmann et al., 2006; Hoover et al., 2020). In agricultural and pastoralist communities across Tanzania, livestock are vital to the economy and daily life. Animals are commonly permitted to roam freely and share communal water sources. Due to limited infrastructure and the lack of designated livestock watering points, humans and livestock frequently use the same water sources (Angelo, 2020). These practices could lead to co-endemicity of *S. haematobium* with *S. bovis* and other trematodes in waterbodies. Additionally, cattle presence could alter waterbodies in ways that indirectly modulate schistosome transmission potential to humans, e.g. by changing nutrient availability or water quality through the deposition of manure. However, this situation contrasts with other communities that have established dedicated livestock watering points, reducing the likelihood of co-endemicity and indirect effects (Moser et al., 2014; Rollinson and Kinung, 2014).

More than 40% of the rural population in Tanzania lacks access to safe water services, while over 60% do not have adequate sanitation facilities (World Bank, 2018). These deficiencies are especially acute in rural areas, where limited infrastructure increases the risk of schistosomiasis as communities frequently come into contact with contaminated water sources. In Zanzibar, reduction of urogenital schistosomiasis has been achieved through different ways, including community-based environmental management through drainage and clearing of vegetation to water contact sites (WHO, 2022). In Tanzania mainland, urogenital schistosomiasis control intervention faces substantial challenges such as the vast scale of waterbody networks, which complicates the intervention strategy. To alleviate the disease burden caused by urogenital schistosomiasis, there is a critical need for precision mapping of urogenital schistosomiasis transmission hotspots and the use of adaptive local strategies tailored to the context of waterbodies at the village level. Significant knowledge gaps remain in understanding how schistosome and non-schistosome trematodes transmission hotspots vary across diverse ecological conditions. Importantly, schistosomes do not exist in isolation within the snail intermediate host. The presence of non-schistosome trematodes in these hosts may lead to interspecific interactions such as facilitation, predation, or competition that could substantially influence schistosome transmission dynamics (Douchet et al., 2024). Elucidating these interactions and spatial differences is critical in identifying disease patterns and optimizing targeted control strategies.

In this study, we comprehensively investigated snail habitats in northwestern Tanzania, revealing how local water use practices contribute to variation in trematode transmission. We specifically (i) assessed presence/absence of snails as well as the prevalence of schistosome and non-schistosome infections in snails in inland waterbodies located in six districts within Lake Victoria areas of Tanzania, (ii) examined the variability in these variables at three spatial scales (waterbody, village, and district), and (iii) evaluated the associations between waterbody characteristics such as size, restriction of cattle use, school proximity and season on these variables. Integrating ecological surveys and mapping techniques sought to uncover the dynamics of *B. nasutus* snail populations and their role in disease transmission, offering critical insights for precision-targeted control interventions of human and livestock parasites for precise and sustainable control and elimination of schistosomiasis.

## 2 Materials and methods

### 2.1 Study areas

**Fig. 1:**
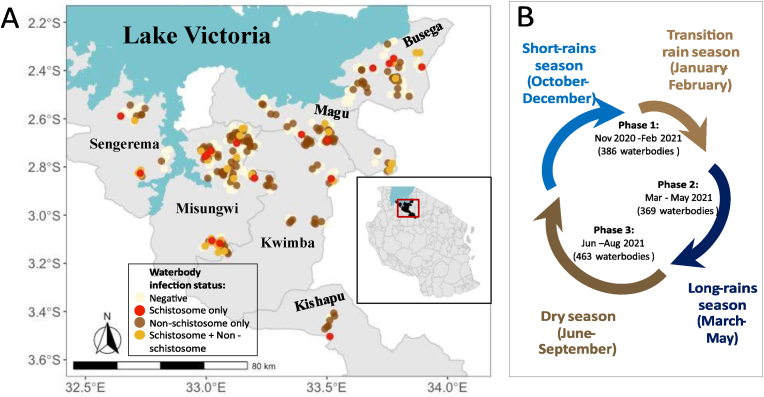
Map of the study area showing waterbodies with snail infection variability (**A**) and seasonal patterns in northwestern Tanzania (**B**).

We collected *B. nasutus* snails from waterbodies in 86 villages across six districts located in three regions of northwestern Tanzania (Fig. 1A). Specifically, sampling occurred in the districts of Kwimba, Misungwi, Magu, and Sengerema within the Mwanza Region, Kishapu District within Shinyanga Region, and Busega District from Simiyu Region. Mwanza region located in the southern part of Lake Victoria shores, had a population of 3,699,872 according to the 2022 national census (DHS, 2022). Mwanza experiences bimodal rainfall patterns, with a short-rains season from October to December and a long-rains season from March to May (Fig. 1). The mean annual rainfall level is between 930 mm and 1200 mm. The region is endemic for *S. haematobium* further inland of the Lake Victoria basin. The predominant species of snail intermediate hosts responsible for urogenital schistosomiasis transmission in the region is *B. nasutus* (Kinoti, 1964; Magendantz, 1972; Rollinson and Kinung, 2014; Gouvras et al., 2017). About 63% of the working population is engaged in small-scale farming (crops and livestock). Farming is predominantly subsistence, undertaken by smallholder farmers with very little commercial inclination in their husbandry practices. According to the 2016 livestock census, the region had a stock of 1,155,871 indigenous cattle, 523,145 goats, and 138,917 sheep. In Mwanza region, Kwimba has the largest share of cattle (34%), followed by Misungwi (21%), Magu (18%), and Sengerema (16%) (Ndesanjo, 2021).

Shinyanga Region is located south of Mwanza Region in the northwestern part of Tanzania and south of Lake Victoria. The region constitutes part of the Lake Victoria Zone and had a population of 2,241,299 according to the 2022 national census (DHS, 2022). The region experiences two rain seasons, a short-rains season between mid-October and December, and a long-rains season between February and May. Shinyanga is a semi-arid region with a mean annual rainfall level of 600–900 mm. Rainfall is inconsistent, unpredictable, and poorly distributed among seasons, leading to a heavy reliance on different waterbodies available for agro-pastoralism, mixing livestock and subsistence farming, exposing populations to *S. haematobium* infections. The region is endemic for *S. haematobium* infections. The snail intermediate host species for schistosomiasis in Shinyanga region is *B. nasutus* (Kinoti, 1964; Angelo, 2020; Civitello et al., 2022). The major activities performed by Shinyanga inhabitants include subsistence farming of food and cash crops; livestock keeping forms a second major economic activity and is part of daily life, which leads to shared waterbodies as watering points and other domestic water usage. The expansion of agricultural fields by clearing forests from the early 1920s to the 1980s has led to the introduction of new snail habitats that contribute to schistosome transmission (Ndesanjo, 2021).

Simiyu Region is located east of Mwanza and Shinyanga regions and southeast of Lake Victoria in northern Tanzania, and had 2,140,497 residents according to the 2022 national census (DHS, 2022). The region experiences two rain seasons, a short-rains season from October to December and a long-rains season from mid-February to mid-May. The mean annual rainfall level is between 700 mm and 1000 mm. The major economic activities of Simiyu inhabitants include small-scale farming and livestock-keeping (UNDP, 2017). The snail intermediate host species for schistosomiasis in Simiyu region is *B. nasutus*. The region is endemic to *S. haematobium* infection, transmitted from temporary and permanent waterbodies present in the region.

### 2.2 Snail sampling

Between November 2020 and August 2021, we surveyed 384 seasonal and perennial waterbodies (ponds) across six districts in northwestern Tanzania (Fig. 1A) to assess the distribution of *B. nasutus* snails and the prevalence of schistosome and non-schistosome trematode parasites. The sampling period was divided into three different phases: November 2020-February 2021 was defined as Phase 1, March-May 2021 as Phase 2, and June-August 2021 as Phase 3 (Fig. 1B). The sampling approach used in this study has been described in detail in previous studies (Hairston et al., 1958). Briefly, this involved searching all waterbodies used by humans and livestock in each village and collecting *Bulinus* spp. snails using a standardized time-constrained sampling procedure (Hairston et al., 1958). Snail abundance was determined through sampling along the edges and on floating objects by two research assistants using hand-held scoops or hand picking for 30 min per site. To ensure a comprehensive representation of the entire water body and capture potential transmission hotspots, sampling was divided into sections to cover the full area within the designated timeframe. If the snail count reached 100 before the end of the sampling period, the collection was concluded early. We characterized the waterbodies surveyed by several criteria: size (length, width, and depth in meters), permanence, distance to the closest school, and permission to access (either by livestock, humans, or a mixture of both livestock and humans). Restrictions on waterbody usage were determined based on the information obtained from the local leadership authority of each village. The location of the school and distribution of waterbodies in each village were determined using a geographical positioning system (GPS), with data taken at every point. Collected snails were placed in clean plastic containers and transported to the National Institute for Medical Research (NIMR), Mwanza Centre, where they were examined for cercarial release to assess active schistosome and non-schistosome transmission.

### 2.3 Snail sample processing and determination of trematode infections

At the NIMR laboratory, snails were cleaned using wet cotton wool and identified to the species level based on their shell morphology, following the guidelines outlined in the identification keys of Mandahl-Barth (1962). *Bulinus nasutus* snails were individually placed in 30-ml shedding beakers filled with 25 ml of bottled water for 24 h under natural light conditions. The beakers were examined for the presence of schistosome and non-schistosome cercariae under a dissecting microscope (Valle et al., 1973). Trematode parasites were split into these two groupings by identifying key morphological traits such as tail shape and movement behavior (Frandsen and Christensen, 1984). In Phase 1, snails were only examined for patent infections with schistosome trematodes assessed *via* cercarial emergence assays. In the course of Phase 1, we anecdotally noted frequent infections with non-schistosome trematodes. Therefore, we also recorded non-schistosome infections in Phases 2 and 3.

### 2.4 Data analysis

We used generalized linear effects models (GLMMs) with binomial error distributions to evaluate the variability in snail presence, schistosome prevalence, and non-schistosome prevalence. We fitted models for snail presence and schistosome prevalence with the full dataset (three phases). However, the non-schistosome model used an abbreviated dataset as snails were only evaluated for non-schistosome infections in Phases 2 and 3. Before considering any predictors, we evaluated variability in snail presence, schistosome prevalence, and non-schistosome prevalence at three spatial scales (district, village, and waterbody). We thus partitioned variance and estimated the proportion of total variance in each endpoint and explained across the three spatial scales by fitting a random effects-only model with random effects of district, village, and waterbody using the *r2* function in the performance package in R version 4.4.1 (Nakagawa et al., 2017). Because the vast majority of variance occurred at the waterbody scale, we then assessed the explanatory ability of several waterbody-specific fixed effects: permanence (permanent *vs* temporary), distance to nearest school (in km, log-transformed), and permission for cattle use (human use only *vs* cattle use permitted). We included Phases 1, 2, and 3 for snail presence/absence and Phases 2 and 3 for non-schistosome models. Additionally, a measure of waterbody size, and the longest dimension (in meters, log-transformed) was only collected in Phases 2 and 3 and was thus only included in the non-schistosome model as a fixed effect. The GLMMs were fitted using the *glmmTMB* function in the *glmmTMB* package in R (Magnusson et al., 2017). In all statistical analyses, waterbodies that were completely dry were excluded. For all snail infection analyses, waterbodies in which no snails were found were also excluded.

## 3 Results

### 3.1 General patterns of snail presence, abundance, and infection prevalence

The distribution of sampling localities is illustrated in Fig. 1A. We investigated 467 waterbodies in 86 villages across six districts in northwestern Tanzania. We found schistosome and non-schistosome infections across all six districts of our study. A total of 43,348 *B. nasutus* snails were collected during the three phases between November 2020 and August 2021 and examined for cercarial emergence of schistosome parasites (Table 1). Of these, 25,052 (57.79%) were also examined for non-schistosome trematode infections between March and August 2021 (Phases 2 and 3). Infection rates of non-schistosome parasites were consistently higher than those of schistosome parasites, regardless of spatial scale. Overall, 0.63% (275 out of 43,348) of *B. nasutus* were patently infected with schistosomes across all three phases, with a median prevalence of 0.08% in Phase 1, 0.10% in Phase 2, and 0.14% in Phase 3. Prevalence increased as the survey entered the dry season in Phase 3. Non-schistosome infections were consistently more prevalent than schistosome infections, and also showed a higher median prevalence in Phase 3 (4.75%) than in Phase 2 (2.74%). Co-infections were uncommon, with only 0.05% of individual snails carrying concurrent patent infections with species of both trematode groups (in Phases 2 and 3). Similar rates of co-occurrence of the two trematode groups in snails from the same waterbody, though not necessarily by the same individual snail, were observed at the district level (Table 1); as a result, no further analyses were run on co-infection or co-occurrence.

**Table 1:** Patterns of snail distribution and abundance, and levels of infection by waterbody and district.

Districts	Busega	Kishapu	Kwimba	Magu	Misungwi	Sengerema	Total
No. of villages (Phases 2 and 3)	18 (17)	3 (3)	4 (4)	13 (13)	27 (27)	7 (7)	72 (71)
No. of waterbodies (Phases 2 and 3)	73 (62)	10 (10)	26 (26)	93 (87)	236 (175)	29 (28)	467 (388)
No. of schools	27	3	4	18	31	9	92
No. of snails (Phases 2 and 3)	6817 (4081)	1617 (786)	2833 (1430)	10,424 (6299)	19,436 (11,289)	2221 (1167)	43,348 (25,052)
Waterbodies with schistosome-infected snails, *n* (%)	8 (11.0)	1 (10.0)	2 (7.7)	7 (7.5)	26 (11.0)	6 (20.7)	50 (10.7)
Snails shedding schistosome cercariae, *n* (%)	108 (1.60)	3 (0.19)	4 (0.14)	15 (0.14)	123 (0.63)	22 (0.99)	275 (0.63)
Waterbodies with non-schistosome-infected snails, *n* (%)	26 (41.9)	7 (70.0)	11 (42.3)	39 (44.8)	86 (49.1)	11 (39.3)	180 (46.4)
Snails shedding non-schistosome cercariae, *n* (%)	177 (4.3)	32 (4.0)	53 (3.7)	374 (5.9)	549 (4.9)	43 (3.7)	1228 (4.9)
Waterbodies where snails are transmitting both parasite groups, *n* (%)	3 (4.8)	1 (10.0)	1 (3.8)	4 (4.6)	15 (8.7)	4 (14.8)	28 (7.2)
Individual snails shedding cercariae from both parasite groups, *n* (%)	1 (0.02)	0 (0)	1 (0.07)	0 (0)	8 (0.07)	2 (0.17)	12 (0.05)

*Notes*: The data obtained in Phases 2 and 3 only are provided in parentheses. For each district, the number of waterbodies and snails singly infected by each parasite group (Phases 1–3 for schistosomes and Phases 2 and 3 for non-schistosomes) or those co-occurring with both parasite groups (Phases 2 and 3) were also quantified. For co-occurrence, waterbody data includes waterbodies where snails are transmitting cercariae from both parasite groups, schistosome and non-schistosome (though not necessarily by the same individual snail).

The results of the random effects-only model for snail presence and infection prevalence of schistosomes and non-schistosome parasites showed the greatest variation at the waterbody scale, with some variation at the district level for snail presence and at the village level for schistosome infection prevalence (Table 2). Specifically, 7.6% of the total variance in snail presence/absence was associated with waterbodies, 2.1% was associated with villages, and 3.3% was associated with districts (Table 2). Schistosome and non-schistosome infection prevalence showed similar associations with waterbody-scale variation (46.6% of total variance for schistosomes *vs* 46.5% for non-schistosomes), whereas infection prevalence for both groups was essentially not associated with district-level variation, and similarly at (∼0% variance at both levels for both parasite groups) (Table 2). Schistosome prevalence was also associated with village-scale variation (28.0% of total variation), while non-schistosome prevalence was not (0%) (Table 2).

**Table 2:** Proportion of total variance (in %) at three spatial scales in random-effects models.

Variable	Spatial scale		
	Waterbody	Village	District
Snail presence	7.6	2.1	3.3
Schistosome prevalence	46.6	28.0	0
Non-schistosome prevalence	46.5	0	0

### 3.2 Factors associated with snail infection at the waterbody scale

**Fig. 2:**
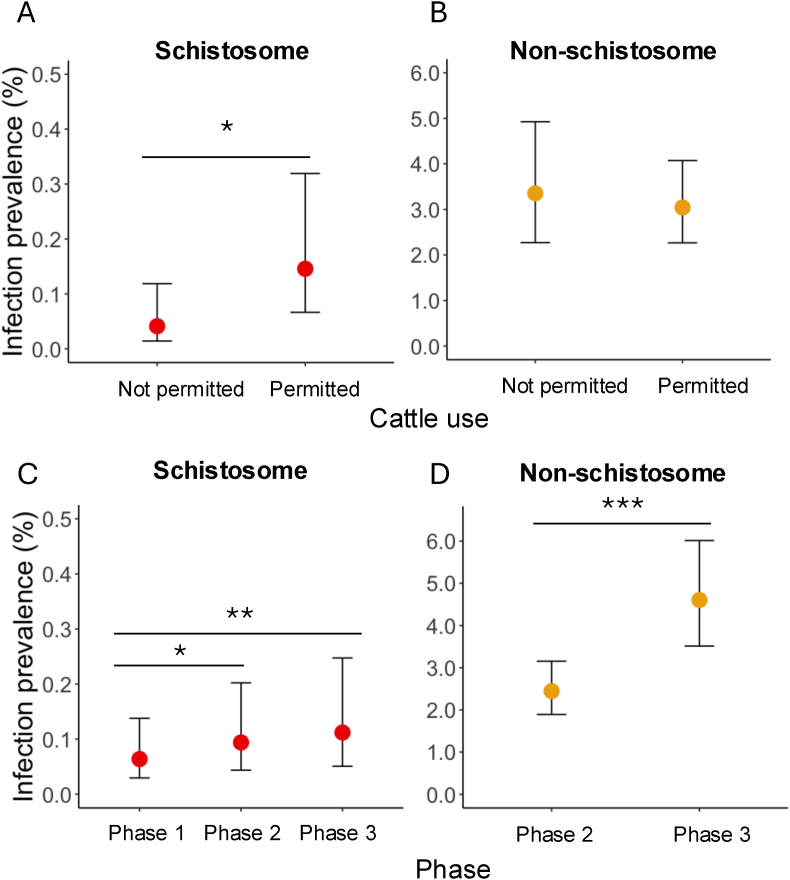
Infection prevalence of schistosomes and non-schistosome trematodes with cattle use permission and across phases. Schistosome infection prevalence was significantly higher in waterbodies where cattle use is permitted than not permitted (*P* = 0.01828) (**A**), whereas there was no difference for non-schistosome trematode infection prevalence (**B**). Both schistosome (**C**) and non-schistosome (**D**) infection prevalence was higher later in the survey period (*P* < 0.05). Note the difference in the range between the figures of schistosome and non-schistosome infection prevalence. Points represent the medians, and whiskers indicate the ranges in prevalence.

**Fig. 3:**
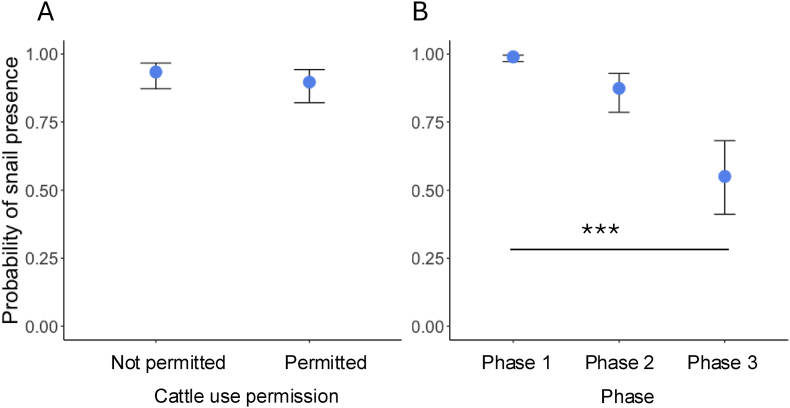
Snail presence/absence with cattle permission and across phases. The probability of snail presence did not vary by cattle use permission (**A**) but decreased significantly (*P* < 0.001) as the survey period progressed (**B**). Points represent the medians, and whiskers indicate the ranges.

The GLMM assessing the explanatory ability of several waterbody-specific fixed effects revealed that schistosome prevalence was significantly higher for waterbodies with permission for cattle use than those for human use only (*P* = 0.01828, Table 3, Fig. 2A). In comparison, non-schistosome trematode prevalence did not vary with permission of cattle waterbody use (Table 3, Fig. 2B). Additionally, schistosome prevalence was significantly lower in Phase 1 than in the two phases that followed (*P* < 0.05 and *P* < 0.01 for Phase 2 and Phase 3, respectively) (Table 3, Fig. 2C). The prevalence of non-schistosome parasites was significantly higher in Phase 3 than in Phase 2 (*P* < 0.0001, Table 3, Fig. 2D) and increased significantly with increasing waterbody size (*P* < 0.01, Table 3). However, the probability of snail presence did not vary by cattle use permission (Fig. 3A) but was significantly lower in Phases 2 and 3 as compared to Phase 1 (*P* < 0.001) (Table 3, Fig. 3B). No other factors were significantly associated with snail presence (Table 3). Neither parasite group (schistosome or non-schistosome) varied in prevalence with waterbody permanence or distance to the closest school (Table 3).

**Table 3:** Binomial error distribution model outcomes for snail presence and infection prevalence of schistosome and non-schistosome trematodes.

Model	Fixed effects	Estimate	*P*-value
Snail presence (Phases 1–3)	Permanence (relative to permanent)	0.13545	0.626957
	Cattle use permission (relative to cattle restricted)	−0.48643	0.074063
	Distance to nearest school	−0.06202	0.725551
	Phase 2 (relative to Phase 1)	−2.60822	9.74e-10∗∗∗
	Phase 3 (relative to Phase 1)	−4.34191	2e-16∗∗∗
Schistosome prevalence (Phases 1–3)	Permanence (relative to permanent)	−0.78428	0.13299
	Cattle use permission (relative to cattle restricted)	1.26667	0.01828∗
	Distance to nearest school	0.05845	0.86511
	Phase 2 (relative to Phase 1)	0.38716	0.03227∗
	Phase 3 (relative to Phase 1)	0.56371	0.00611∗∗
Non-schistosome prevalence (Phases 2 and 3)	Permanence (relative to permanent)	0.06833	0.77094
	Cattle use permission (relative to cattle restricted)	−0.10047	0.67599
	Distance to nearest school	0.01584	0.91697
	Phase 3 (relative to Phase 2)	0.65496	2e-16∗∗∗
	Maximum waterbody length	0.28965	0.00627∗

*Note*: ∗*P* < 0.05, ∗∗*P* < 0.01, ∗∗∗*P* < 0.0001.

## 4 Discussion

We investigated the distribution and abundance of snails and their patent schistosome and non-schistosome trematode infections across a heterogeneous landscape in northwestern Tanzania. In our survey spanning six districts and 86 villages across three regions, we found that snails, schistosomes, and other trematode parasites are highly variable at smaller spatial scales, i.e. particularly at the waterbody scale. This suggests that specific activities, preferences, or water uses determine which waterbodies harbor snails infected with schistosome or non-schistosome parasites. We found a positive association between schistosome infection and cattle use permission. This could indicate a direct role of cattle through the presence of *S. bovis* or hybrids (Oyeyemi et al., 2023; Ame et al., 2025) or indirect effects of cattle on *S. haematobium* transmission, through dung deposition or by influencing human patterns of water contact. Similarly, the association between waterbody size and non-schistosome parasites could indicate a direct effect of waterbody permanence or indirect mediation of social factors or behaviors that depend on waterbody size. These patterns suggest that the risk of transmission of schistosome and non-schistosome parasites is highly localized to waterbody characteristics.

The high variability of schistosome prevalence at the waterbody scale has implications for the prevention of exposure and transmission control. First, it suggests that villages contain some waterbodies that are transmission hotspots and others with no transmission potential. We documented this variation within a year. However, multi-year studies are critical to determine if transmission hotspots persist through longer periods or are formed sporadically. If transmission hotspots are relatively stable, then focused interventions could be directed at a subset of waterbodies, which might be cost-effective. For example, snail control or behavioral modification programmes might be directed at waterbodies that permit cattle use, resulting in greater transmission potential. While it remains unclear if the waterbodies are transmission hotspots for livestock or human trematodes, mitigation efforts, such as preventing dung deposition, could disrupt *S. haematobium* and *S. bovis* life cycles by reducing schistosome egg input (of the latter) and limiting the introduction of organic material that could subsidize large snail populations.

Designation of waterbodies for domestic use and cattle use may serve to reduce human contamination of domestic water sources, as indicated by the significantly lower schistosome infection in domestic water sources. Previous studies have documented that some trematodes, such as echinostomes, feed on and kill sporocysts of subordinate species, thus offering direct competition to subordinate species and eliminating prior infections in snail hosts, preventing the establishment of the subordinate species (Kuris, 1987). This indicates that the lower schistosome infections might be contributed to by different factors, including the presence of non-schistosome species in snail intermediate hosts. Community members enforce maintenance to keep waterbodies used for domestic and drinking water free from human and cattle excreta contamination. The specific rules and level of enforcement differ from community to community based on water availability and demand, or knowledge of waterborne diseases. Other studies have found that limiting waste disposal to waterbodies serves as an alternative strategy for water-borne disease control, such as schistosomiasis (Fenwick et al., 2006; Tchuenté et al., 2012). Thus, the targeted and consistent use of distinct waterbodies for distinct purposes could be a key behavioral intervention to reduce transmission of schistosomiasis until the broad availability of adequate water and sanitation measures (Freeman et al., 2014). Alternatively, the elevated prevalence of schistosome parasites in cattle-permitted waterbodies could suggest that the schistosome parasites we observed in our study could be *S. bovis* or *S. haematobium* or *S. haematobium* × *S. bovis* hybrids, directly using cattle or other livestock as definitive hosts, demonstrating competence of *Bulinus* snail vectors for multiple schistosome species, including hybrids (Webster et al., 2013). For example, a recent study in Benin identified cattle as potential definitive hosts for *S. haematobium* and *S. bovis* (Savassi et al., 2020).

However, we would then also expect an elevated prevalence of non-schistosome parasites, which are likely predominantly infecting cattle in cattle-permitted waterbodies. The equivalent prevalence of non-schistosome parasites regardless of cattle permission suggests that while humans may avoid contamination of cattle-restricted waterbodies, cattle regulations are not well adhered to, or that these waterbodies still receive eggs of these parasites in runoff. In practice, cattle in pastoral communities are not strictly managed; they are left wandering around even during flooding periods, which can lead to direct introduction or runoff into sites deemed as “restricted” (Savassi et al., 2020). Spatially heterogeneously distributed infection of cattle and humans with schistosome parasites has been reported in other studies (Greter et al., 2017), as well as in Tanzania, a previous study reported prevalence of infection and diversity of trematodes in *B. nasutus* varied considerably between habitats and by season, with identification in a few habitats (Loker and Gardner, 1981). Therefore, there is a growing need to pair ecological surveys, such as ours, with molecular identification techniques (Kane et al., 2008; Tumwebaze et al., 2019; Crego-Vicente et al., 2021) to shed light on the transmission dynamics of trematode parasites infecting humans, livestock, and wildlife within a One Health approach. Molecular identification of parasites or snails was not conducted in this study, limiting the clear identification of the prevailing schistosome parasites being transmitted in the study area. Furthermore, systematic studies examining interspecific competition between *Schistosoma* spp. and other trematodes in *Bulinus* hosts are warranted, as such interactions may modulate local transmission potential. Resolving the genetic and species identities of the parasites we observed here could reveal direct interactions within snail vectors, e.g. dominance hierarchies (Laidemitt et al., 2019), or clarify how coexisting parasite taxa differentially adapt to shared ecological gradients (Haas, 2003).

While the prevalence of non-schistosome parasites did not vary with cattle permission, non-schistosome prevalence increased with waterbody size. This is likely since larger waterbodies are used by more cattle, serve as cattle gathering stations, and are less likely to dry up, thus maximizing transmission in the later season. Counter to our hypotheses, waterbody permanence and proximity to school were not significantly associated with snails or infections. We expected permanent waterbodies to be more suitable for snails and to facilitate human and animal access year-round, thereby having greater infection rates. Similarly, we expected that waterbodies near schools would be important exposure sites (Savioli et al., 2004). The lack of association with school distance indicates that other activities may be more important, e.g. swimming, fishing, bathing, fetching water, searching water for watering livestock, and washing clothes are among risk practices for schistosome transmission (Angelo et al., 2018, 2019; Mereta et al., 2019). Indeed, swimming during the weekend (i.e. non-school days) represents a substantial portion of water contact time for children (Person et al., 2016). Therefore, identification of specific transmission hotspots within a complex transmission network can serve as an alternative strategy for schistosomiasis control (Angelo et al., 2018).

We found that snails were more likely to be present early in the survey period preceding the harsher waterbody conditions associated with heavy rains (Phase 2) and the dry season (Phase 3). We did not identify any other factors explaining snail occurrence. Other studies have demonstrated that schistosome-vectoring snails can be associated with water parameters, such as ionic concentration (Dida et al., 2014), water permanence and flow rate (Perez-Saez et al., 2016), aquatic vegetation (Wood et al., 2019), and presence or absence of predators (Allan et al., 2020). In contrast, the prevalence of schistosome and non-schistosome parasites decreased across the phases of the study as snail presence decreased, suggesting seasonal trends in trematode transmission (Sturrock et al., 2001), similar to previous studies (Lwambo, 1988; Woolhouse and Chandiwana, 1988; Sturrock et al., 2001; Phiri et al., 2005; Greter et al., 2017). As the dry season progresses, snail populations decrease due to the contraction of waterbodies and the onset of snail aestivation (Senghor et al., 2015). They also decrease due to increased disturbance caused by elevated human and livestock use (Kinoti, 1964; Okeke and Ubachukwu, 2013). The increase in parasite prevalence could reflect the passage of adequate time for the trematodes to complete their development and emerge from snail hosts. It could also reflect increased parasite input into these waterbodies as humans and livestock increasingly use fewer and fewer waterbodies, as many become fully dry. Year-round studies with finer temporal resolution are needed in highly seasonal landscapes such as this inland region of northwestern Tanzania to determine these seasonal patterns as well as to assess how fluctuations in abiotic factors such as temperature, rainfall, and water velocity influence snails and trematode transmission dynamics (Rollinson et al., 2001; McCreesh and Booth, 2013; Kalinda et al., 2017b). Future studies would also benefit from the identification of prepatent schistosome infections in snails. This might provide a more accurate measure of the prevalence of schistosome and other trematode infections in the waterbodies, as our patent infection detection assay potentially underestimates infection rates; missing the prepatent stages of schistosome infections also affects identification of co-infections with *S. haematobium* and *S. bovis.*

While we found little variation among villages or districts in snail presence or infection prevalence, we did not assess human, livestock, or wildlife infections. Therefore, our results do not imply that villages or districts do not vary substantially in the human or animal burden of infection or disease. Discrepancies in transmission between villages suggest that variations in water contact patterns are likely important. For example, household proximity to foci of snail vectors (Paredes et al., 2010; Angelo et al., 2018) and lack of local access to safe waters causes inevitable contact with infested water, leading to high transmission (Kabatereine et al., 2014). Therefore, our findings of waterbody-scale transmission factors can complement other studies across larger scales to illuminate transmission hotspots within a network of complex transmission that can guide the development of targeted interventions for schistosomiasis control and elimination.

## 5 Conclusions

The study demonstrates a distinct seasonal peak in *B. nasutus* schistosome infections during the dry season (June-August). Waterbodies permitted for cattle use showed a higher prevalence of schistosome infections in *B. nasutus* than waterbodies used only by humans. Non-schistosome trematode parasites remained consistent across all spatial scales. This observation suggests that the integration of snail control during the dry season for schistosomiasis control is critical. Cattle may play an indirect role in schistosome transmission, necessitating integrated interventions focusing on cattle and human management in the current schistosomiasis control programs. The maintenance of non-schistosome parasites in all waterbody types indicates widespread co-endemicity, highlighting for targeted interventions across waterbody usage with a focus on the role played by cattle in schistosome transmission.

## CRediT authorship contribution statement

**Teckla Angelo:** Conceptualization, Methodology, Investigation, Data curation, Writing – original draft, Writing – review & editing. **Naima Camilla Starkloff:** Investigation, Data curation, Visualization, Writing – original draft, Writing – review & editing. **David James Civitello:** Investigation, Conceptualization, Visualization, Writing – original draft, Writing – review & editing. **Moses Paul Mahalila:** Investigation, Methodology, Writing – review & editing. **Safari Kinung’hi:** Conceptualization, Investigation, Methodology, Validation, Writing – review & editing.

## Ethical approval

The study received ethical approval from the Medical Research Coordination Committee (MRCC) of the Tanzania National Institute for Medical Research (NIMR), ethics approval (certificate number NIMR/HQ/R.8a/Vol.IX/3462).

## Funding

This study was supported by the United States of America National Institute of Allergy and Infectious Diseases (R01 AI50774-01 and D43TW011813).

## Declaration of competing interests

The authors declare that they have no known competing financial interests or personal relationships that could have appeared to influence the work reported in this paper.

## Data Availability

All data generated or analyzed during this study are included in this published article. Raw data can be accessed online at https://github.com/naimastarkloff/PilotProjectTanzania.
